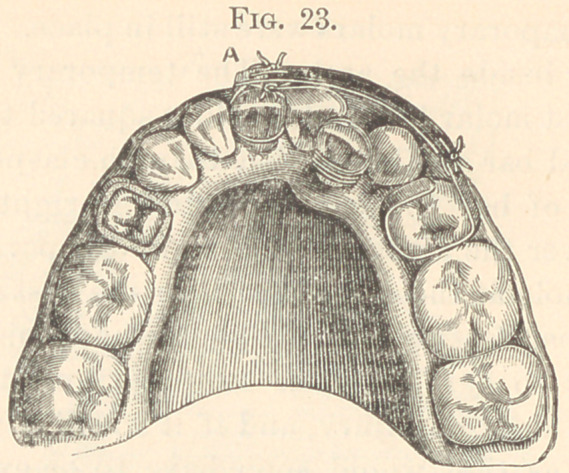# Regulators and Methods of Correcting Irregularities

**Published:** 1889-02

**Authors:** W. G. A. Bonwill

**Affiliations:** Philadelphia


### REGULATORS AND METHODS OF CORRECTING IRREGULARITIES.

BY W. G. A. BONWILL, D. D. S., PHILADELPHIA.

(CONCLUDED.)

I have had the greatest satisfaction in the use of gutta-percha on the proximal surfaces of the temporary molars, which, as long as it can be kept in them, spreads the jaws or keeps the permanent molars from crowding forward.

The trouble has always been to get hold of any of the temporary teeth, as a fulcrum.

It has been my practice for years first to make use of the silk igature and rubber bands without plates. To do so, how shall the ligature be prevented then from slipping off the permanent tooth, or from slipping down over the temporary tooth, which is being used as the fulcrum.

I argue that, as the temporary cuspids and first molars will soon be lost after the permanent lateral incisors have come and are high enough to get hold of, it is well to cut a slot with a small hard-rubber disk on their buccal and palatal surfaces deep enough to hold the ligature, which keeps it from ever passing down under the gum, Fig. 22. If a plate of rubber or metal must be made for the inside, use the same grooves to hold the plate in position.

If a clasp is needed, which is most frequently the case in the use of the new appliance, presently to be shown, cut the first temporary molar on its mesial and distal surfaces, a little under parallel, as in Fig. 23, and the strain is so slight that it is not uplifted before the lateral incisor has been drawn into the circle. If there is any danger from the ligature wounding the gum, place gutta-percha underneath. If I want to pass a ligature around a perma-

Fig. 12.

nent tooth (Figs. 12 and 17) as a fulcrum, I simply warm a small piece of gutta-percha and press it on the palatal or lingual side of the tooth, letting it extend slightly down on the gums, and when cold remove, cut two holes to let the ligature pass through it, and then between the teeth, and tie outside to the rubber band. This little adjunct cannot be overpraised ; for it is so soon adjusted, is pleasant to the patient and non-irritating to the tissues. If a metal wire or band is pressing into the gums, and a hook cannot be used on the grinding surface, the gutta-percha fills the need :

and it answers well as a fulcrum by letting the band directly into the gutta-percha or by attaching it to the wire or silk ligature that holds the former.

The lower jaw partially shows the application of the gutta-percha stay-plate (see Fig. 11) for keeping the ligature off the gum at the crevix, on the first molar. The lower jaw in this case (see Fig. 12) being too large an arch for the upper, I extracted the first right bicuspid; and as the right lateral inferior incisor was too far in the arch, and the right cuspid very far outside, I simply ligated the first inferior molar on the same side. A piece of pink base-plate gutta-percha was warmed and pressed up against the molar, letting it rest partially on the adjoining teeth (see Fig. 11); when cold, two holes were made in it for the passage of the ligature, which was tied on the buccal surface of the molar. A rubber band was tied to the inside before adjusting. A ligature was then cast around the right lateral, carried up between it and the cuspid, and over it through the space where the first bicuspid was extracted, on the lingual side of the first bicuspid, and tied to the rubber band attached to the gutta-percha stay or helmet on the first molar, and stretched over the buccal surface of the cuspid. This drew the lateral out very forcibly. The ligature was lastly placed on the cuspid alone, and remained for six weeks without change. The same appliance shown in Fig. 11 is also applied in Fig. 17. This was a very contracted lower arch with a deep underbite. The arch was first expanded by the fixture shown in Fig. 13, made of piano wire, with half clasps

Fig. 11.

Applied in Figs. 12 & 17.

Fig 13.

Applied to Fig 17—Expander of bicuspids, lower.

Fig. 14.

Shown as applied, Fig. 15.

of platinized gold at A A, made with small ears to rest on the grinding surfaces of the first bicuspids to prevent slipping down upon the gums. These clasps were soft-soldered to retain the full temper of the piano wire as a spring. It is a very cheap and easy way of making such an apparatus and with a powerful spring which such cases demand.

Fig. 15.

In this case I could not afford to extract any teeth, because the incisors were already touching the gums on palatal side of the superior centrals. In expanding the lower arch I obviated this deep over and underbite. The left lateral was very far inside the arch, and the cuspid so far as nearly to allow the bicuspid to touch the lateral. The silk ligature was first placed over the lateral and carried up next the cuspid. The first bicuspid was ligated with a stay-plate or helmet of gutta-percha on its lingual side with the ligature running through both holes and carried around the first bicuspid and tied on the buccal side. This prevented entirely the slipping of the ligature upon the crevix. A rubber band was then stretched between the lateral and the bicuspid and secured. This expanded the arch in front and drew out the lateral very quickly. These little gutta-percha caps or helmets work admirably, and are not displaced in mastication.

Fig. 14 is another modification of Fig 10, the single bar, and is applied in Fig. 15, where the four superior incisors are to be moved forward from one-fourth to three-eights of an inch and the whole arch expanded to meet the more perfect and larger arch in the lower. It is made of two flat bars of platinized gold sliding over each other for at least two inches. A loop is soldered to the end of each flat bar as guides to hold them in place while sliding through. A rubber band is shown attached to the end of each bar at AA, which, in contracting, enlarges the circle, and consequently not only throws out the incisors, but the bicuspids and cuspids as well.

Fig. 16.

The attachments are made on either side to a molar or a bicuspid, owing to the ease of clasping. I have utilized the decay on the anterior surface of a molar by filling with amalgam, and cutting a hole for one end of the bar to rest in instead of using a clasp.

The apparatus is shown applied in Fig. 15, with the bars some distance away from the incisors to be moved.

Before the apparatus is placed permanently in position, the four incisors are ligated with a loop, as shown in Fig. 22, using gum sandarach varnish to prevent slipping or turning on the tooth. The ligature should be so adjusted as to twist the tooth, if needed, while drawing it forward. These are then tied to the sliding bars, bringing them closely in contact with all the teeth in the arch. The rubber band is then tied between the two points AA, and the application is complete. It is easy to see not only its simplicity, but also its great effectiveness. It can be used equally well for contracting an arch.

Fig. 17.

Fig. 18 shows the worst case of protrusion of the upper jaw I have ever seen. It did not arise from an acquired habit, nor did it have any precedence in heredity. The temporary teeth had proper arches. No cause could be assigned. They came as you

Fig. 18.

see in Fig. 18. The lower incisors, when I first saw the case, were three-eighths of an inch from the superior incisors on their palatal surface, and were imbedded in the gums on the hard palate.

Before attempting to draw in the incisors I made a rubber plate (Fig. 19) to cover the hard palate, thickened where the lower teeth would touch, and opened the jaws at the bicuspids at least one-eighth of an inch. This was not only to drive the inferior

Fig. 19.

incisors up into their sockets, but also to allow the bicuspids and molars to come down and antagonize before the plate was removed. Two years were consumed in this. To this plate was then attached a rubber band carried entirely around the arch with a silk ligature, and a metal hook, with two holes, was carried over the cutting edge of the central incisors, through which the ligature passed. This kept the ligature down on the incisors near the cutting

Fig. 20.

edges, and while it was aiding in drawing in the arches, it did another important thing: forced the centrals up into the alveolus. This was done by the tendency of the rubber band to work up towards the gums, and at the same time it pressed them up and made them shorter without grinding. This was a case parallel with the one delineated by Dr. Kingsley in "Oral Deformities," but without any of the treatment given there. The sliding band in Fig. 14 would have done well here, but I adopted the simpler one of ligature and rubber. A gold band, running over the arch from the second bicuspids, which was soldered to clasps around the latter, and which could be adjusted or removed by the patient, was used to secure it in position.

The rubber plate was removed as soon as I began to draw the incisors into the arch, to allow them to adapt themselves to the smaller arch. Fig. 21 shows the application of the band to the lower

Fig. 21.

jaw where the temporary molars were still in place. The permanent laterals were far inside the arch. The temporary cuspids also remained. The first molar had had its sides squared to retain a clasp. A platinized gold bar similar to Fig. 10, with clasps, was used with holes at the end of bar C., and opposite the right central incisor with another over the centre of the right temporary cuspid at G, and the fourth hole at the end of bar near the first molar at B.

The principal feature about this method, aside from the bar, was the cutting so heroically the temporary molars for retaining the plate. This does no injury, and if it was likely to would make little difference, as they would soon have to be extracted in order to make room for the bicuspids.

The ligatures are applied, as in all cases where this bar has been used, so as to press backward as well as drawing outward. In this case two separate pieces of rubber band were used.

Fig. 22. The feature about this case which made it novel and unique was the utilization of the superior temporary cuspids for holding the ligature. To place a ligature on the temporary teeth insures their removal or extraction without this plan. To keep the ligature on the body of the tooth, I take a small hard-rubber corundum disk and make a groove on both the labial and palatal sides of the cuspid, deep enough for the ligature to rest securely. If necessary, I should cut the first or second temporary molars if a ligature could be gotten around the incisors to be turned into place.

Fig. 22.

The rubber band was drawn through between the centrals, which gave it more power over the incisors. The left superior lateral was soon placed in the arch.

Fig. 23 shows the cut surface in the first temporary molar on the left and the tooth on the right with the clasp around it attached to the bar. The ligature passes between the lateral over

FIG. 23.

the central and through the hole in bar at A, pressing the central to the left and the molar to right.

The utilization of the temporary molars, in cutting them for clasping, or by grooving for ligatures, cannot be overestimated. They thus form fulcrums, which may be utilized early in the treatment of the case, which heretofore has baffled our skill. The sooner we begin to correct irregularity the easier and more complete our result.

The explanation of geometrical law, and the value of the anatomical articulation in showing how the first permanent molar plays so important a part in making the lower incisors roll over one another, and thus make a smaller arch with a very deep under and overbite where seen. I am almost quite ready to say, *never* extract the first permanent molar. Keep down the inferior incisors. Have the permanent molar take its place soon and rapidly in the arch. Drive it backward toward the ramus rather than have it move forward to make the underbite too deep.

To a person of any comprehension these are simple devices and plain rules; the application can be made to any case of irregularity. Any one can surely make the apparatus. Whoever hereafter shall undertake this branch of practice should first read my article on the geometrical law of articulation and study the principles involved, and not attempt wildly to do what but few men have ever truly fathomed. Really, in every city, some one should make of this a special practice, and the profession should encourage such by sending cases for his inspection and consultation. And such a specialist should do all he can in return to teach by example and demonstrations by clinics, to enlighten those who are placed so far from large cities that they are compelled to take such cases. When we can have that understanding between us, then we may feel as banded brothers more fully equipped for these hitherto difficult and almost thankless operations.

## Figures and Tables

**Fig. 12. f1:**
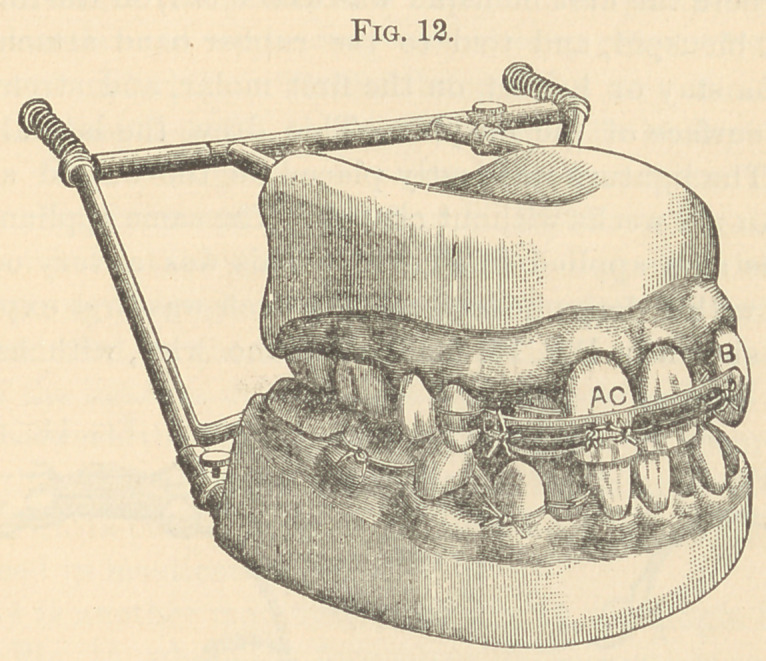


**Fig. 11. f2:**
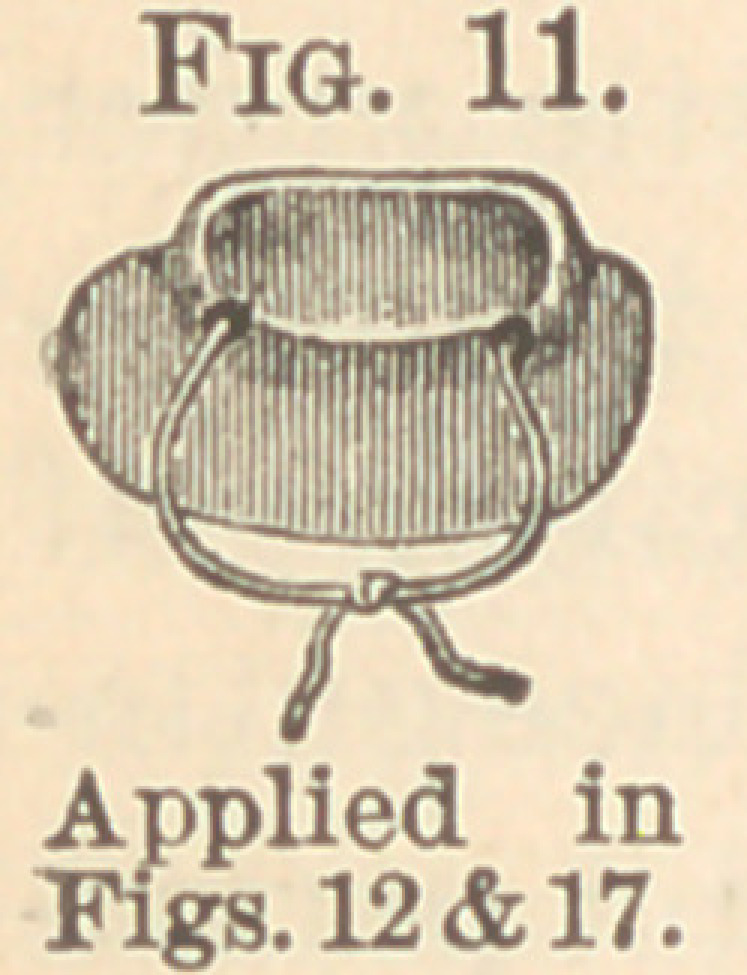


**Fig 13. f3:**
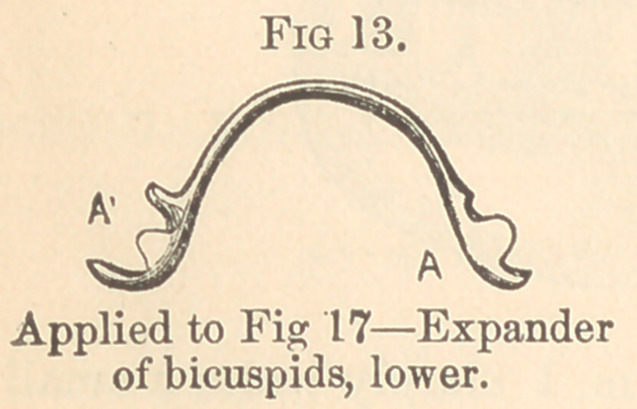


**Fig. 14. f4:**
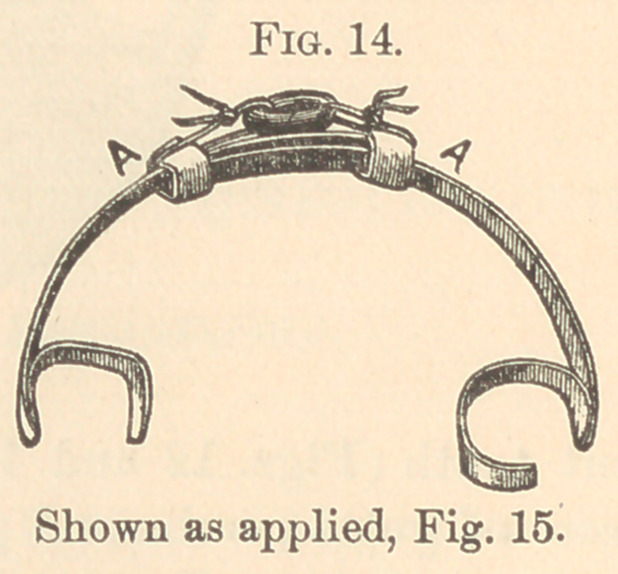


**Fig. 15. f5:**
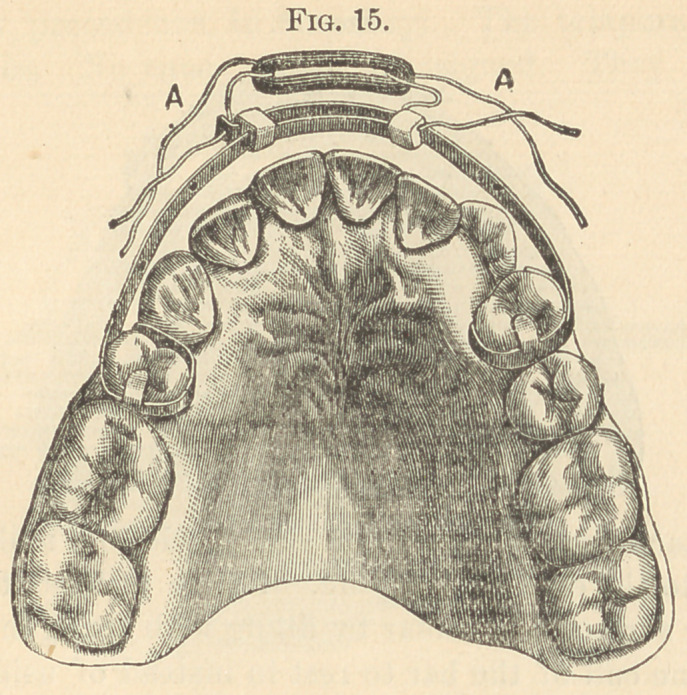


**Fig. 16. f6:**
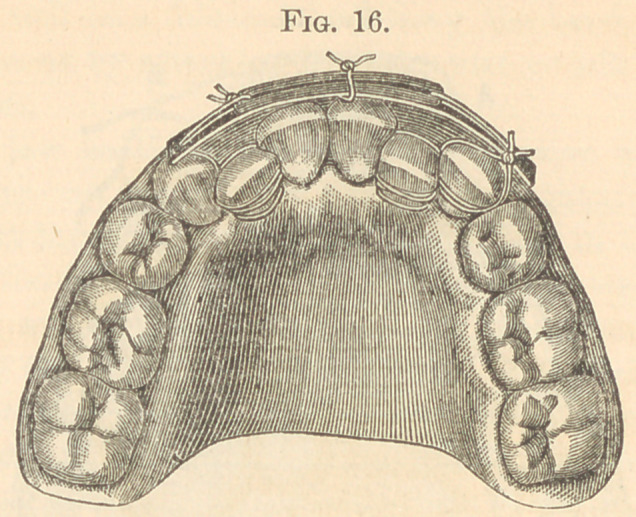


**Fig. 17. f7:**
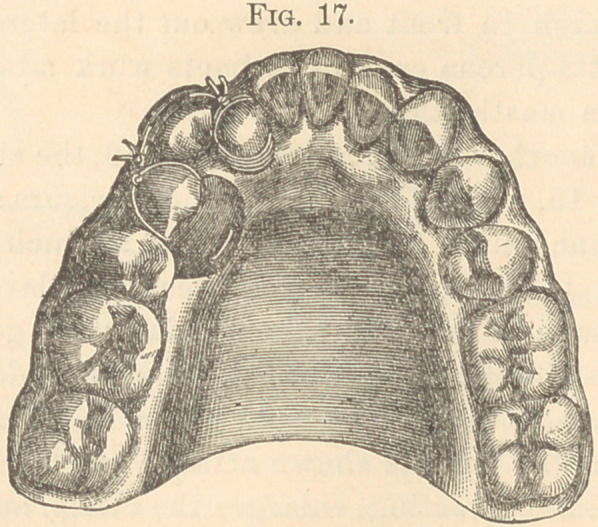


**Fig. 18. f8:**
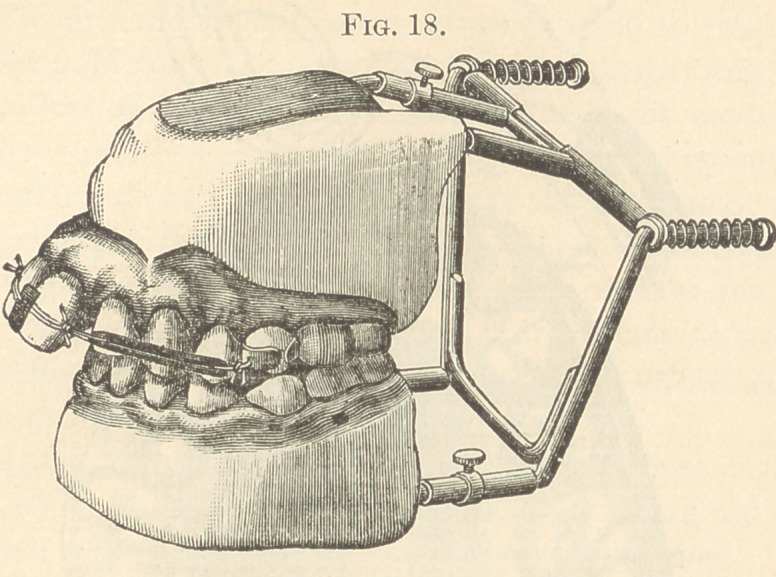


**Fig. 19. f9:**
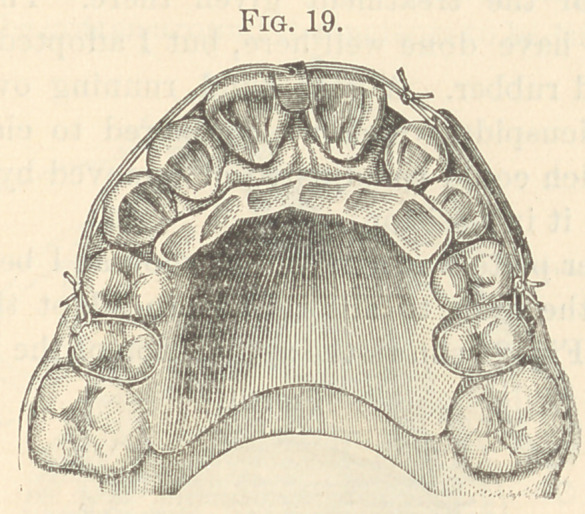


**Fig. 20. f10:**
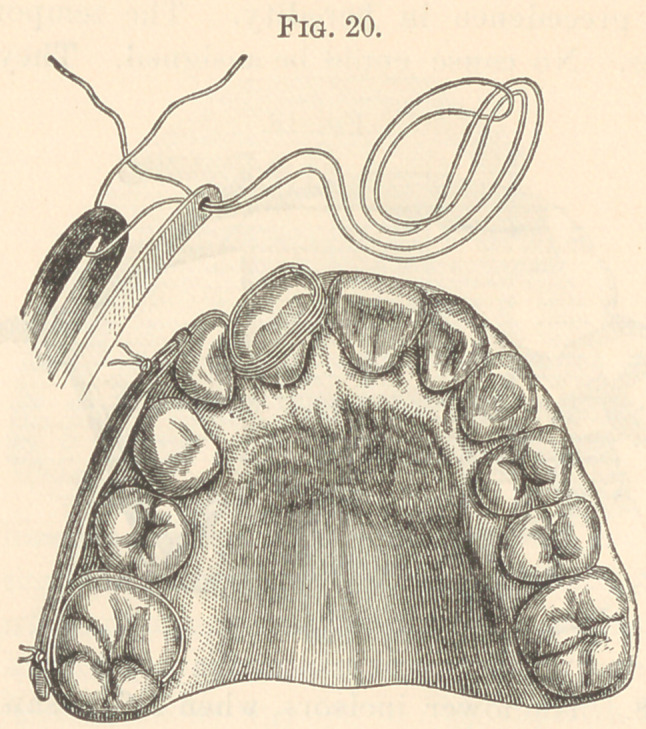


**Fig. 21. f11:**
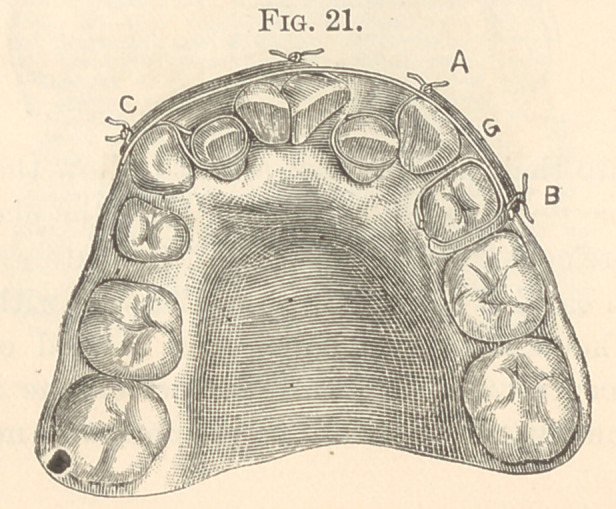


**Fig. 22. f12:**
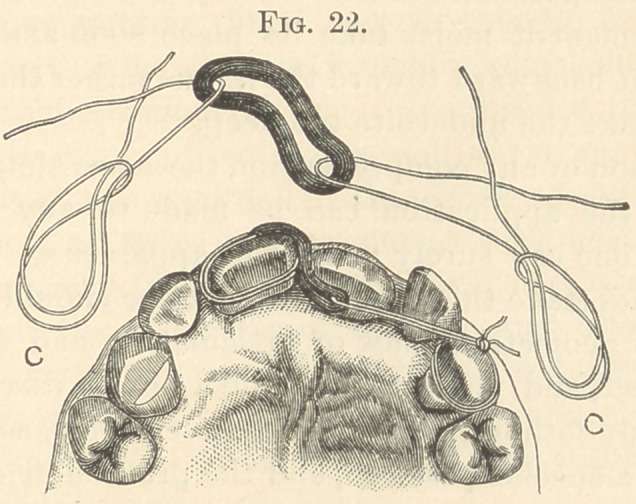


**Fig. 23. f13:**